# Liquid Fraction Effect on Foam Flow through a Local Obstacle

**DOI:** 10.3390/polym14235307

**Published:** 2022-12-05

**Authors:** Oksana Stennikova, Natalia Shmakova, Jean-Bastien Carrat, Evgeny Ermanyuk

**Affiliations:** Lavrentyev Institute of Hydrodynamics, 630090 Novosibirsk, Russia

**Keywords:** foam rheology, liquid fraction, Hele-Shaw cell

## Abstract

An experimental study of quasi-two-dimensional liquid foams with varying liquid fractions is presented. Experiments are conducted in a Hele-Shaw cell with a local permeable obstacle placed in the center and filling 35, 60 and 78% of the cell gap. Foam velocity is calculated using a standard cross-correlation algorithm. Estimations of the liquid fraction of the foam are performed using a new simplified method based on a statistical analysis of foam cell structures. The pattern of the foam velocity field varies with increasing liquid fraction, responsible for significant variation of the foam’s rheology. The local permeability decreases with increasing obstacle height and liquid fraction. In case of high liquid fraction ($5.8\times 10^{-2}$), the permeability coefficient tends to zero for obstacles filling more than 78% of the cell gap.

## 1. Introduction

Foam is a complex, two-phase medium behaving like a non-Newtonian fluid: at low applied stress, it exhibits elastic properties, and at a stress above a certain threshold value, it can flow like a viscous liquid [1,2]. Due to their specific properties, foams have a large range of applications from household, such as cosmetics and food, to industry, where it is used for the creation of various porous materials [3] or oil production [4,5]. In the latter case, the use of foams can significantly reduce the volume of water during hydraulic fracturing and minimize negative environmental impact. It has been shown recently that biocompatible foams have great potential for creating new photobioreactors providing a higher density of biomass compared with other methods [6]. Introducing polymer materials into foams leads to the development of new porous materials that are used in aircraft components, sports and medical equipment [7]. New methods for producing porous polymers, such as the solution foaming strategy, have been proposed [8]. By varying the key parameters, it is possible to obtain foam cell structures with hexagonal or circular shapes [9] and control the cell distribution [10]. For example, morphology of the liquid crystals used as electro-optic materials depends on the liquid-crystal fraction that controls the amount and size of “bubbles” in the final product [11]. Finally, a method for synthesizing macroporous polymers was proposed using aqueous foams [12]. The behavior of two-dimensional aqueous foams is the main subject of this article.

In all the abovementioned applications, understanding the mechanisms that allow for the control of foam flows turns out to be fundamentally important.

One of the key parameters of the foam is the liquid fraction, which is the volume of liquid per unit volume of foam. The liquid fraction affects local processes at the level of individual bubbles and, as a consequence, the macroscopic properties of the foam. In particular, drier foams have a higher yield stress compared with more liquid foams [1,2]. At a certain critical value of water content (≈36% for three-dimensional foam and ≈9% for two-dimensional foam), there is a transition from foam flow to the so-called bubble flow, where the bubbles are practically not connected to each other, and the medium loses the ability to sustainably maintain its structure [13,14]. Local topological rearrangements at the level of individual bubbles (T1 events) occurring when the yield stress surpasses a certain critical value define the plasticity of the foam [15]. Such rearrangements lead to plastic deformation and viscous energy dissipation and have a significant effect on the flow structure [16,17]. Combining the elastic, plastic and viscous behavior of foam into a single model remains an open question to this day [2,18].

Quasi-two-dimensional geometries are often used to study foam flow [19,20], offering the possibility to observe local internal processes experimentally via optical methods. In this article, we use a version of a quasi-two-dimensional setup to study the effect of liquid fraction on the key parameters and patterns of the foam flow around an obstacle. Indeed, there is a lack of pertinent experimental data obtained from a wide range of liquid fraction values. Our goal is to fill this gap in a systematic way as required for better understanding of the foam rheology and its intrinsic relation with observed flow patterns and topographic rearrangements of the foam structure. The progress in this direction has been hindered by the absence of an efficient algorithm for automatic evaluation of the water content in the foam based on the in situ experimental images. Below, we describe the experimental setup and propose a new method of automatic evaluation of the liquid fraction from optical images in Section 2. The experimental results are presented and discussed in Section 3 and summarized in Section 4.

## 2. Materials and Methods

### 2.1. Experimental Procedure and Data Processing

**Figure 1:**
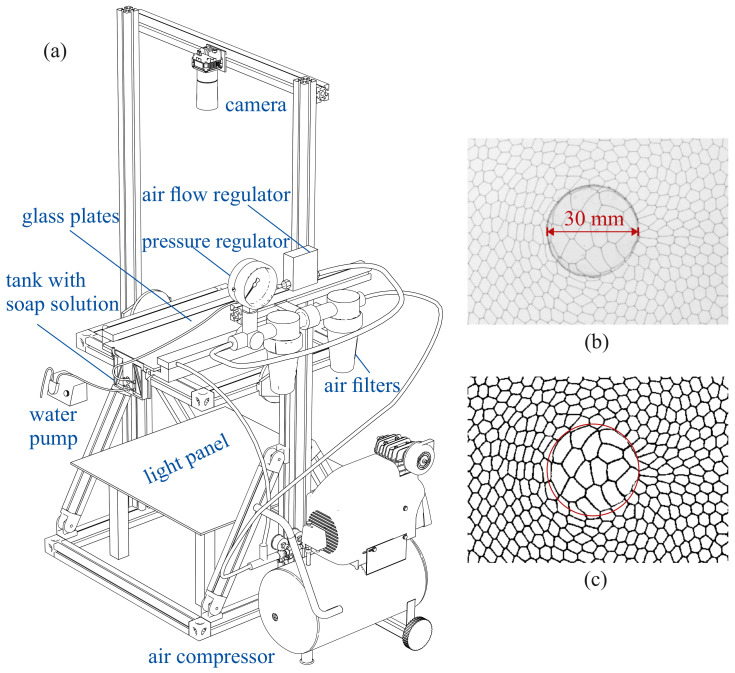
(**a**) Experimental setup: a Hele-Shaw cell consisting of two glass plates with a gap of G=1.15 mm, the tank (in situ foam generator) attached to the bottom plate with holes for air and fluid injection, the light source below the cell and the camera above it; the air from the compressor is partially used for pressing the glass plates; compressed air passing filters enters the air flow regulator and the tank via the needle. The water pump controls the soap solution flow rate. (**b**) Raw image of the foam flow zoomed in on the permeable obstacle area (obstacle diameter a=30 mm) and (**c**) its binarization, with the red circle corresponding to the position of the permeable obstacle.

Quasi-two-dimensional foam flow is generated in a Hele-Show cell (Figure 1) consisting of two horizontal parallel glass plates with dimensions 640×230mm separated by a gap of height G=1.15 mm. The working area of width 180 mm is limited by a silicon joint on the sides, preventing leakage. A small tank with a height of 90 mm filled with an 18% solution of a surfactant (${\mathrm{Fairy}}^{\mathrm{TM}}$) in distilled water is attached to the bottom plate on one side of the cell while the opposite end remained free (open) to ensure the foam flow. The upper plate of the cell is placed between metal bars with silicon tubes filled with compressed air to ensure an equal pressure along the cell, squeezing the sides of the upper plate to longitudinal spacers to ensure the uniformity of the gap height *G*. A local permeable obstacle represented by a flat disk with a diameter of a=30 mm cut from plastic of thicknesses H=0.4, 0.7 and 0.9 mm (respectively, 35, 60 and 78% of the gap height *G*) is glued to the bottom plate in the center of the working area. The upper edge of the disk is rounded to avoid destruction of the foam bubbles when they pass the obstacle.

The foam is generated in situ in the small tank by injecting the pressured air in the soap solution at a constant rate $F_{a}=1.0\;\mathrm{mL}/s$ through a needle with an inner diameter of 0.45 mm. According to Fritz’s formula [21], the needle diameter determines the diameter of the foam bubbles, which remains constant in all experiments $d_{bubble}\approx$ 3.9 mm. The air supply is provided by an air compressor through a filter system and is controlled using an analog gas supply regulator RRG-12. The liquid fraction of the foam varies by either increasing the level of soap solution *h* in the tank or co-injecting the soap solution using a pre-calibrated INTLLAB DP-385 dosing pump while the tank is filled to the top (90 mm). The level of the solution in the tank is h=45, 70 and 90 mm. At higher *h*, the foam has a higher liquid content since it travels a shorter distance from the liquid surface to the top of the tank before entering the Hele-Show cell [22]. The flow rate of the co-injected soap solution (at h=90 mm) is 0.16 and 0.27mL/min. Thus, five different values of liquid fraction Φ are considered in this paper $\Phi =3.8\times 10^{-3},4.7\times 10^{-3},1.0\times 10^{-2},3.7\times 10^{-2}$ and $5.8\times 10^{-2}$, which are calculated using the algorithm described below in Section 2.2.

When the air is injected in the tank containing the soap solution it creates excessive pressure, forcing the foam to move through the working area. Since the size of the foam bubbles in the foam generator exceeds the size of the gap *G*, the bubbles are deformed at the inlet of the cell, thereby creating a monolayer foam of thickness 1.15 mm, which has a quasi-two-dimensional structure. The transparency of the cell allows the video registration of the foam flow using a camera JAI SP-20000M with a resolution of 5120×3840 pixels (see the zoomed image example in Figure 1b). The camera is fixed above the Hele-Show cell illuminated from below by the LED panel, as shown in Figure 1. The camera frame rate is fixed at 4 frames per second in all experiments. The spatial resolution evaluated from the size of the obstacle in the frame is 12.5pixel/mm and the size of the working area is $320\times 192\;{\mathrm{mm}}^{2}$. The duration of each experiment is 125 s, corresponding to 500 frames. A video of the liquid foam flow ($\Phi =1.0\times 10^{-2}$ and H/G=0.6) from the foam generator to the free end of the cell is available as Supplementary Material Video S1.

The recorded images are first binarized using ImageJ (see Figure 1c), which allows the analysis of individual white areas corresponding to bubbles (foam cells), including their geometry and mutual arrangement. The velocity field is obtained by cross-correlation of the successive binarized images with PIVlab—the Time-Resolved Digital Particle Image Velocity Toolbox for MATLAB [23]. The Cartesian coordinate system is introduced with its origin in the center of the obstacle, the *x*-axis in the direction of the foam flow and the *y*-axis perpendicular to it in the plane of the cell. The linear dimensions are normalized by the obstacle diameter *a* (e.g., Figure 3). We obtain the two-dimensional velocity fields U=(u,v) averaged in time over the entire duration of each video recording (125 s), where *u* is the longitudinal velocity component along the foam flow (in *x* direction) and *v* is the transverse component (in *y* direction).

### 2.2. Computer Method for Liquid Fraction Calculation

In this paper, we refer to the effective liquid fraction defined in [13], which coincides with the liquid fraction in the case of an ideal two-dimensional foam. The definition of the effective liquid fraction is based on the assumption that all the liquid is concentrated at the Plateau borders and the liquid contained in the films separating the bubbles can be neglected [13]. The liquid fraction of the foam is thus determined by the ratio between the area occupied by the liquid and the total area occupied by the bubbles including their walls. The area occupied by the liquid $A_{\mathrm{PB}}$ is related to the radius of curvature of the Plateau borders *R* as $A_{PB}=\left(\sqrt{3}-\pi /2\right)/R^{2}$ [1]. Taking into account that each Plateau border is shared between three foam bubbles and that each bubble has on average six “neighbors” [13], we obtain $A_{\mathrm{liquid}}=6A_{\mathrm{PB}}/3$. The radius of curvature *R* can be related to the critical distance between adjacent bubbles $L_{c}$ before the bubble rearrangement or T1 event as $R/\sqrt{3}=L_{c}/2$ (see Figure 4 in [13]). Thus, the liquid fraction can be obtained by the following formula [13]:

(1) $$\Phi =\frac{3}{2}\left(\sqrt{3}-\frac{\pi }{2}\right)\frac{L_{c}^{2}}{A_{\mathrm{bubble}}}\approx 0.242\frac{L_{c}^{2}}{A_{\mathrm{bubble}}}.$$

Hence, to determine the liquid fraction of the foam the critical distance $L_{c}$ and the average foam bubble area $A_{\mathrm{bubble}}$ should be found.

Previously [13,24], the value of $L_{c}$ was obtained based on manual measurements “by eye”, which is a time-consuming process responsible for large errors in calculations. Several methods are used to detect T1 events, including the use of neural networks [25,26]. Here, we propose a simple algorithm to obtain $L_{c}$ based on a purely statistical approach. To improve the statistical analysis, we select a part of an image downstream of the obstacle where most of T1 events occur. We also remove all the bubbles adjacent to the boundary of the cropped image by filling them with black color so that they do not contribute in the average bubble area calculation. Using the standard functions of the MATLAB Image processing toolbox package, we estimate the area and identify the center of each bubble represented by a domain of white pixels in the binarized image.

**Figure 2:**
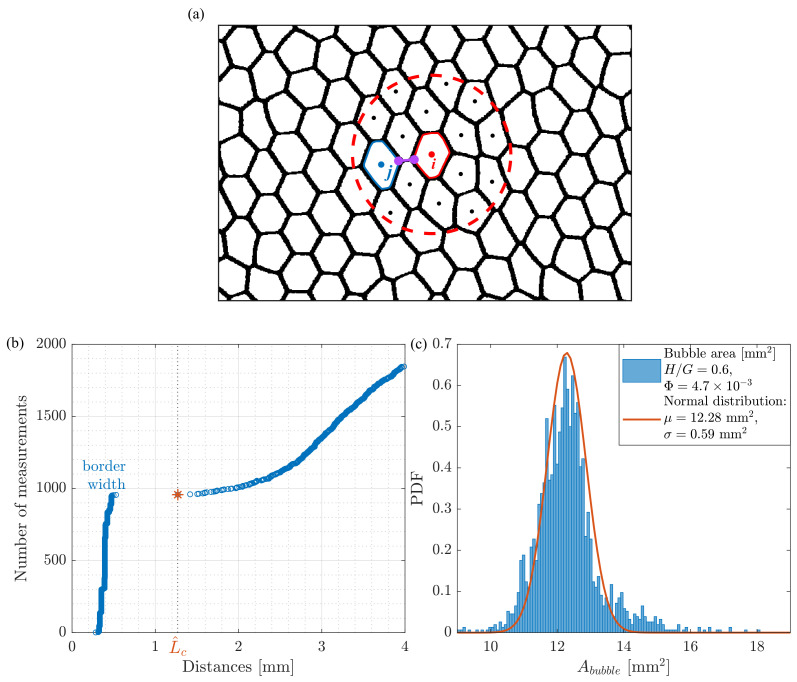
Method used to find the critical distance $L_{c}$ before rearrangement (or T1 event) occurs: (**a**) selection of a bubble and all its neighbors, which are located at a distance less than $2d_{mean}$ from its center; (**b**) an example of sorted distances between the foam cells in the ascending order, the red star indicates ${\hat{L}}_{c}$—the critical distance plus border width; (**c**) histogram of bubble areas superposed with a Gaussian fit (Normal distribution with $s=12.28\;{\mathrm{mm}}^{2}$ and $\sigma =0.59\;{\mathrm{mm}}^{2}$).

For subsequent image processing, we first select one bubble (*i* in Figure 2a) and search for all bubbles whose centers lie at a distance of no more than two average bubble diameters ($d_{mean}$), as shown in Figure 2a, where the area of interest is delimited by the red dashed line. Further, we estimate the distances between all combinations of points lying on the borders of the white areas between two bubbles, for example, between the solid red and blue contours, respectively, for the bubbles *i* and *j* (purple line) in Figure 2a. To reduce the computational effort, we estimate this distance using every 50-th image. Afterwards, we sort the distribution of this distance in ascending order, as shown in Figure 2b. The distances between the cells are divided into two groups: the width of the black border between adjacent bubbles and the distances between “neighbors”. Between these two groups, there is a sharp jump in the value just before it corresponds to $L_{c}$ plus the border width (red star and ${\hat{L}}_{c}$ in Figure 2b). To obtain the correct critical distance $L_{c}$, we simply subtract the average width of the black border between adjacent bubbles from the obtained value.

Note that the $L_{c}$ value has an error due to the spatial resolution. The standard deviation for the width of the cell boundary is 0.05 to 0.15 mm, giving an error for $L_{c}$ measurements from 0.13 to 0.23 mm. Taking into account that the $L_{c}$ value ranges from 0.5 to 2.2 mm, the relative error ranges from 10% to 26% for various experiments, which is a considerable improvement compared with manual processing.

While executing the image processing, we simultaneously estimate the average bubble area ($A_{bubble}$) based on the number of pixels that belong to each bounded white area. The result of the analysis based on 10 frames with an interval of 12.5 s is shown in Figure 2c.

The above-described method works for dry and liquid foam flows up to the critical liquid fraction value when the air bubbles become virtually independent (bubble flow) and no $L_{c}$ can be calculated. In the later case, simple measurement of the volume of liquid coming out of the Hele-Shaw cell might be used to calculate the liquid fraction.

**Figure 3:**
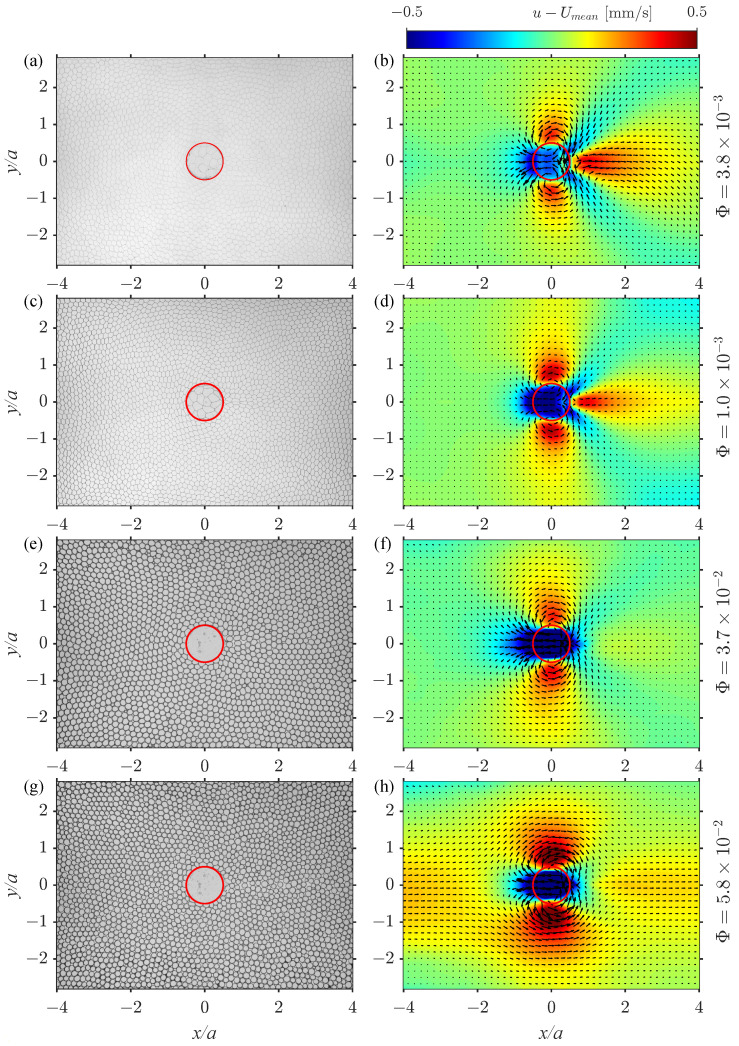
Original images of the foam (on the left) and longitudinal velocity component averaged over 125 s with subtracted mean velocity $u-U_{mean}$ (color) together with the velocity vector field $U-U_{mean}$ (on the right) for various liquid fraction Φ: (**a**,**b**) $3.8\times 10^{-3}$, (**c**,**d**) $1.0\times 10^{-2}$, (**e**,**f**) $3.7\times 10^{-2}$ and (**g**,**h**) $5.8\times 10^{-2}$.

Figure 3 (left column) demonstrates the data for H/G=0.6 and the liquid fractions $\Phi =3.8\times 10^{-3},1.0\times 10^{-2},3.7\times 10^{-2}$ and $5.8\times 10^{-2}$, where the values of Φ are estimated via Equation (1) from $L_{c}$ determined with the help of the above-described procedure. It can be seen that for the two smallest liquid fractions (see Figure 3a,c) the foam cells have straight borders. With increasing Φ, the amount of liquid in Plateau borders increases and the areas filled with air become more circular. Raufaste et al. [13] define the critical value $\Phi =9\times 10^{-2}$ when the foam flow transforms to liquid bubble flow, where each air bubble has a circular shape and can move independently. Note that the diameter of such a bubble (seen from above) is roughly three times larger than the gap height *G*. In this work, we reach values very close to $\Phi =5.8\times 10^{-2}$ and observe a similar bubble behavior.

## 3. Results

### Velocity Field

Let us discuss the structure of the time-averaged velocity field U measured at different values of liquid fraction Φ of the foam. To better visualize the key qualitative effects, we subtract from the original data the mean velocity $U_{mean}$ (the longitudinal velocity component averaged in space over a rectangular area upstream of the obstacle where the velocity field is virtually unperturbed and represents a uniform inlet flow):

$$U_{mean}={\langle u\rangle }_{x\in [-5,\;-4],y\in [-3,\;3]}.$$

Note that this area spans over the whole width of the flow. Figure 3 (right column) shows the longitudinal velocity component $u-U_{mean}$ (in color) and the velocity vector field U for various liquid fractions of the foam and a fixed height of the obstacle H/G=0.6. For small liquid fractions $O(10^{-3})$, the flow upstream of the obstacle is uniform and corresponds to the mean flow $U_{mean}$. We observe a dipolar recirculation of the flow close to the obstacle sides (in the transversal direction) and a so-called negative wake [27] or an overshoot downstream of the obstacle (Figure 3b,d) where the velocity is higher than the inlet velocity $U_{mean}$. These results are in agreement with [13,28] for a non-permeable obstacle and [24] for a permeable one. In case of a permeable obstacle, the negative wake is a consequence of the release of the energy stored in the deformed foam bubbles when they pass over the obstacle.

**Figure 4:**
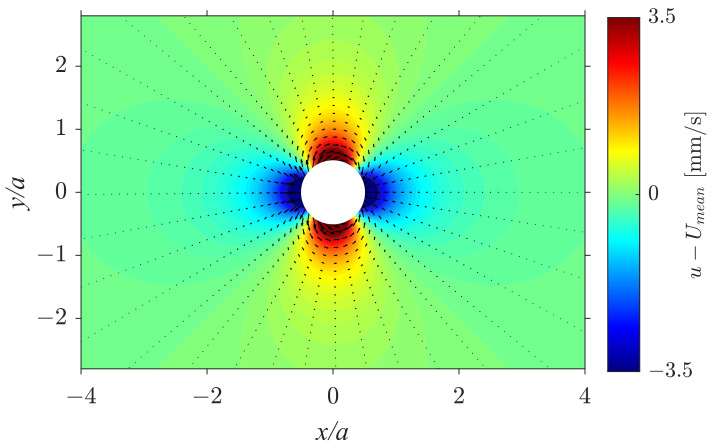
Analytical calculation of a two-dimensional potential flow passing a circular obstacle with the longitudinal velocity $u-U_{mean}$ in color and the vector field $U-U_{mean}$ shown with black arrows.

With increasing liquid fraction, the flow becomes more symmetrical with respect to the obstacle in the *x* direction (Figure 3f). When the liquid fraction approaches the critical value for transition to a bubble flow regime ($\Phi =5.8\times 10^{-2}$) the inlet flow is no longer uniform along the *y* direction. This is due to the injection of the air and the fluid at one single location (y=0) in the foam generator. While the dry foam fills entirely the working area in the *y* direction and its velocity is uniform, the bubble flow appears to be faster in the vicinity of the centerline of the cell (y=0), where the injection takes place. The approximate symmetry of the bubble flow with respect to the *x*- and *y*-axes justifies a comparison with potential flow around a cylinder, which describes the streamlines of the flow of Newtonian fluid in a sufficiently narrow Hele-Shaw cell. The irrotational flow around a cylinder without circulation has the following form:

$$\begin{array}{c} V_{r}=U_{mean}\left[1-{\left(\frac{a/2}{r}\right)}^{2}\right]\cos\theta ,V_{\theta }=-U_{mean}\left[1+{\left(\frac{a/2}{r}\right)}^{2}\right]\sin\theta , \end{array}$$

where θ= and *r* are cylindrical coordinates (x=rcosθ,y=rsinθ), $V_{r}$ and $V_{\theta }$ are the radial and angular velocities, and $U_{mean}$ is the imposed velocity from the experiment. Thus, in the Cartesian coordinates, the velocity components are $u=-\sin\theta V_{\theta }+\cos\theta V_{r},v=\sin\theta V_{r}+\cos\theta V_{\theta }$. Figure 4 demonstrates the longitudinal velocity component $u-U_{mean}$ and the velocity field $U-U_{mean}$, with U=(u,v). It can be seen that this pattern qualitatively corresponds to the pattern shown in Figure 3h for $\Phi =5.8\times 10^{-2}$, implying qualitative similarity between the bubble flow and the flow of Newtonian fluid.

**Figure 5:**
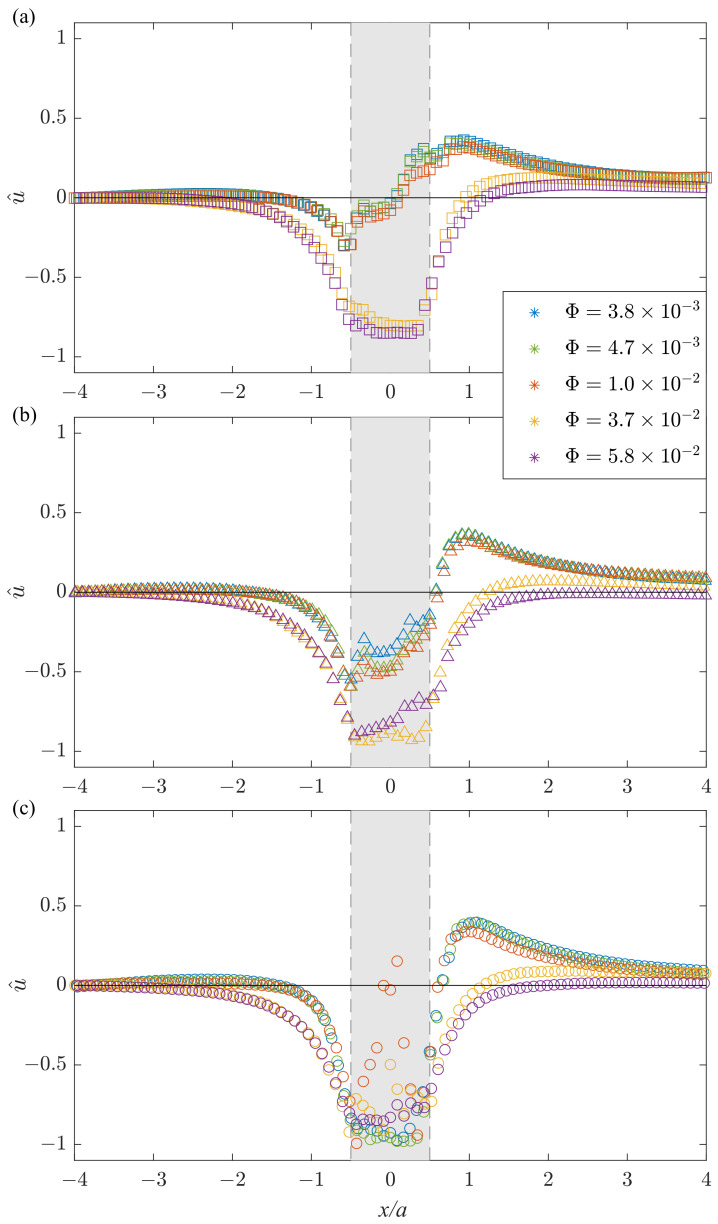
Profiles of the normalized longitudinal velocity $\hat{u}$ along the *x*-axis for obstacle heights H/G of (**a**) 0.35, (**b**) 0.6 and (**c**) 0.78 and liquid fractions of $\Phi =3.8\times 10^{-3}$ (blue), $4.7\times 10^{-3}$ (green), $1.0\times 10^{-2}$ (red), $3.7\times 10^{-2}$ (yellow) and $5.8\times 10^{-2}$ (purple).

Let us now discuss the evolution of the flow pattern with the obstacle height and the liquid fraction in terms of the normalized longitudinal velocity component

$$\hat{u}=\frac{u-U_{mean}}{U_{mean}}$$

along the *x*-axis. Figure 5a–c show the profiles of $\hat{u}$ for the obstacles with heights H/G=0.35,0.6 and 0.78, respectively, where each color corresponds to different liquid fractions $\Phi =3.8\times 10^{-3},4.7\times 10^{-3},1.0\times 10^{-2},3.7\times 10^{-2}$ and $5.8\times 10^{-2}$.

**Figure 6:**
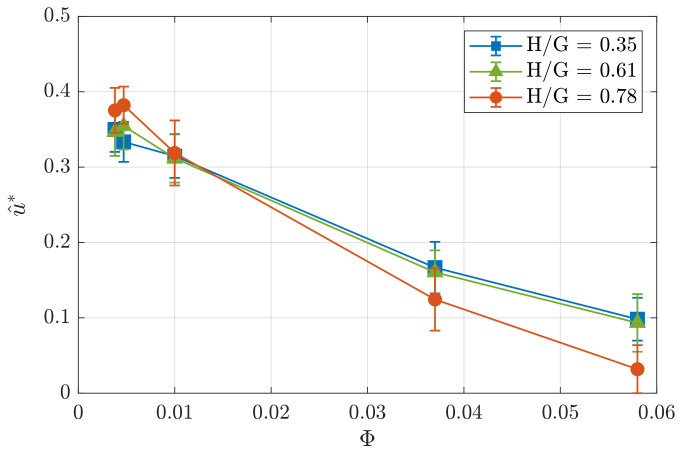
Maximum normalized longitudinal velocity ${\hat{u}}^{*}$ with respect to the foam liquid fraction for H/G=0.35,0.6 and 0.78.

For higher obstacles, the velocity overshoot increases in agreement with the results described in [24]. However, with increasing liquid fraction, the velocity overshoot decreases differently for various object heights. For H/G=0.35 and $\Phi =5.8\times 10^{-2}$, one may still observe the asymmetry of the flow up- and downstream of the obstacle (Figure 5a), while for H/G=0.78 and the same liquid fraction the profile of the normalized longitudinal velocity $\hat{u}\left(x/a\right)$ becomes nearly symmetrical (Figure 5c). In the case of drier foams, the energy stored in deformed bubbles passing over the obstacle has a major influence on the local increase in velocity (overshoot) due to the elastic properties of foam bubbles. In case of the foam with high liquid fraction, when the gap decreases (i.e., at high obstacle height), the probability of events when a bubble may pass over an obstacle decreases due to a dramatic decrease in the tension from the neighboring bubbles. At the same time, the probability of topological rearrangements of bubbles (change of neighbors) in the foam flow around the obstacle increases, leading to higher viscous energy dissipation and decrease in velocity, with complete suppression of the overshoot effect. To illustrate this in more detail, we plot separately the velocity maxima ${\hat{u}}^{*}$ for three obstacles (H/G=0.35, 0.6 and 0.78) as a function of the liquid fraction in Figure 6. It can be seen that with an increase in the liquid fraction, the maximum velocity decreases. For H/G=0.35 and 0.6, the decrease is roughly linear, while for H/G=0.78, a second- (or higher-) order polynomial function is required to provide a reasonably close fit. The decrease in the maximum velocity with increasing percentage of the liquid in the foam occurs due to the decrease in the yield stress of the foam. As a result, foams with higher liquid fraction are subject to higher deformations (local rearrangements) when passing around obstacle. Similar behavior is qualitatively predicted by the theoretical model [29].

**Figure 7:**
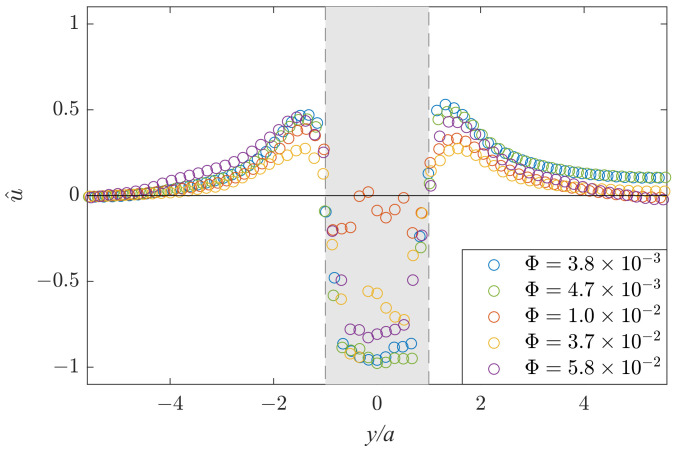
Profiles of the normalized longitudinal velocity $\hat{u}$ along the *y*-axis for the obstacle height H/G=0.78 and the liquid fractions $\Phi =3.8\times 10^{-3}$ (blue), $4.7\times 10^{-3}$ (green), $1.0\times 10^{-2}$ (red), $3.7\times 10^{-2}$ (yellow) and $5.8\times 10^{-2}$ (purple).

An important characteristic of the flow is the permeability factor of the local obstacle proposed in [24], which determines how much the flow rate through the obstacle area decreases in relation to the flow rate in the absence of any obstacle. The permeability factor *Q* is defined using the longitudinal velocity component $\hat{u}$ along the *y*-direction (x=0) shown in Figure 7.

In the experiment with the highest liquid fraction ($\Phi =5.8\times 10^{-2}$) and the obstacle height H/G=0.78, the foam flow structure is qualitatively similar to the classic viscous Hele-Shaw flow. This suggests that the regime of the liquid bubble flow around the obstacle is reached while the bubble flow in the constricted area above the obstacle becomes negligible. The other limiting case of dry foam corresponds to the foam moving as a whole (as a sort of tissue), excluding the region of deformations (which are weak at low H/G) due to the presence of the obstacle. The observed flow patterns are between these two limiting cases of low and high permeability.

**Figure 8:**
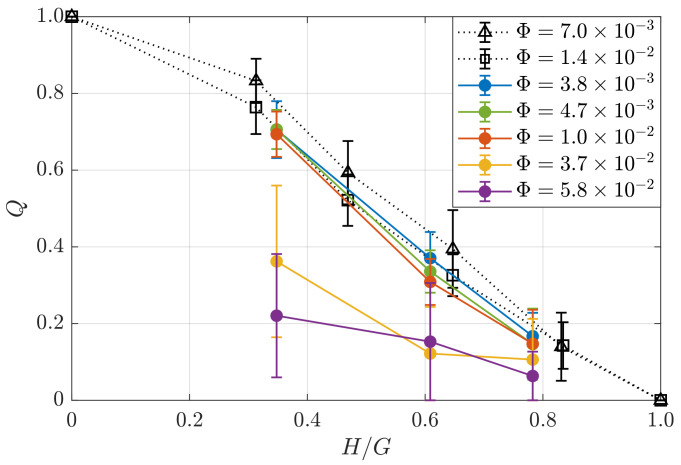
Permeability factor *Q* calculated using Equation (2) with respect to the normalized obstacle height H/G for liquid fractions $\Phi =3.8\times 10^{-3}$ (purple), $4.7\times 10^{-3}$ (yellow), $1.0\times 10^{-2}$ (red), $3.7\times 10^{-2}$ (green) and $5.8\times 10^{-2}$ (blue) and the data from [24] for $\Phi =7.0\times 10^{-3}$ (black triangles) and $1.4\times 10^{-2}$ (black squares).

The permeability factor *Q* is calculated as follows [24]:

(2) $$Q=\frac{l}{a}-\frac{\int_{-l/2}^{-a/2}udy+\int_{a/2}^{l/2}udy}{aU_{mean}},$$

where *l* is the width of the working area, *a* is the diameter of the obstacle and dy is the width of the cross-correlation algorithm windows. The permeability factor is shown in Figure 8 as a function of the normalized object height H/G for various values of the liquid fraction. For the foams with low liquid fraction ($\Phi <10^{-2}$), the results are close to those obtained in [24]. With increasing H/G and/or higher liquid fraction Φ, the permeability of the area delimited by the perimeter of the obstacle decreases. In particular, for the obstacle filling 78% of the gap and the liquid fraction $\Phi =5.8\times 10^{-2}$, the permeability parameter becomes nearly zero. As it has been already mentioned, under these conditions, the air bubbles tend to bypass the obstacle instead of passing over it due to the (i) dramatic decrease in the yield stress, (ii) increased probability of local rearrangements and (iii) high energy barrier required to squeeze a bubble to allow it to pass through the constriction. These results confirm the assumption of [24] that at a certain critical value of the liquid fraction, the permeability parameter will drop virtually to zero, even for permeable obstacles. In our experiment, this critical liquid fraction value is $\Phi =5.8\times 10^{-2}$.

## 4. Conclusions

This article presents experimental results for foam flow in the Hele-Shaw cell with a local permeable obstacle in the range of liquid fractions from $3.7\times 10^{-3}$ to $5.8\times 10^{-2}$. We first present a simple automatic method to obtain the value of liquid fraction in the foam based on the statistical analyses of the binarized experimental images. Based on the results obtained by this method, we describe the foam flow with different liquid fractions in terms of its velocity field measured with the cross-correlation algorithm. We observed the following:

- An increase in water content decreases the effect of a negative wake for the foam flowing around an obstacle, with full suppression of the effect in bubble flows;
- For drier foams, an increase in the obstacle height leads to an increase in the effect of a negative wake;
- The permeability of the local obstacle decreases significantly with an increase in the liquid fraction in the studied range of parameters and drops to a value of nearly zero for the liquid fraction above $5.8\times 10^{-2}$. The existence of a critical liquid fraction corresponding to the near-zero permeability factor at a permeable constriction is important for efficient flow control in applications related to the production of polymer materials based on aqueous foams.

## Data Availability

The data that support the findings of this study are available from the corresponding author upon reasonable request.

## Supplementary Materials

The following supporting information can be downloaded at: https://www.mdpi.com/article/10.3390/polym14235307/s1, Video S1: The liquid foam flow (liquid fraction $\Phi =1.0\times 10^{-2}$ and normalized obstacle height H/G=0.6) from the foam generator to the free end of the Hele-Shaw cell.
