# Supplementary figures and images for: Liquid Fraction Effect on Foam Flow through a Local Obstacle
Source: Polymers (Basel). 2022 Dec 5;14(23):5307. doi: 10.3390/polym14235307 (PMC9739718; doi:10.3390/polym14235307)

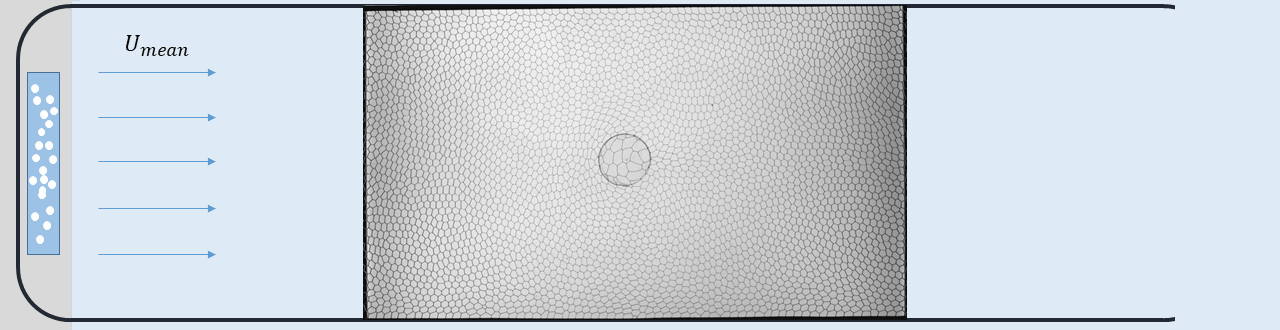

Supplement: Supplementary file 1 [file polymers-14-05307-s001.zip › supplementary video S1.gif]
